# Primary Adrenal Insufficiency, Complete Sex Reversal, and Unique Clinical Phenotype in a Patient with Severe CYP11A1 (P450scc) Deficiency—Case Report and Literature Overview

**DOI:** 10.3390/children11101231

**Published:** 2024-10-12

**Authors:** Zuzanna Nowak, Ewelina Preizner-Rzucidło, Jakub Gawlik, Jerzy B. Starzyk, Dominika Januś

**Affiliations:** 1 Hospital of the Brothers Hospitallers of Saint John of God, 31-061 Krakow, Poland; zuzanna.nowak@alumni.uj.edu.pl; 2 Department of Pediatric and Adolescent Endocrinology, Institute of Pediatrics, Jagiellonian University Medical College, 30-663 Krakow, Poland; jerzy.starzyk@uj.edu.pl; 3 Department of Genetics, Institute of Pediatrics, Jagiellonian University Medical College, 30-663 Krakow, Poland; e.preizner-rzucidlo@uj.edu.pl; 4 University Hospital, 30-688 Krakow, Poland; jagawlik@su.krakow.pl; 5 Department of Pediatric and Adolescent Endocrinology, University Children Hospital in Krakow, 30-663 Krakow, Poland

**Keywords:** CYP11A1, P450scc, primary adrenal insufficiency, sex reversal, 46, XY

## Abstract

Background: Congenital adrenal hyperplasia (CAH) is a group of genetic disorders that lead to the dysfunction of the steroidogenesis pathway, resulting in steroid hormone deficiency of varied intensity. The cholesterol side-chain cleavage enzyme (P450scc), coded by the *CYP11A1* gene, is vital to the first step in the biosynthesis of steroid hormones, which is the conversion of cholesterol to pregnenolone. Therefore, its deficiency causes a general steroid hormone shortage. Objective: We report a case of CAH caused by P450scc deficiency with complete 46, XY sex reversal, characteristic facial features (narrow middle section of the face, small ears with thick helix, fleshy upturned lobules), and dysmorphic macrocephaly along with shortened upper and lower extremities. Results: Our patient carries a compound heterozygotic pathogenic variant of the *CYP11A1* gene, with two frameshift pathogenic variants NM_000781.3(*CYP11A1*):c.358del (p.Arg120Aspfs*18) in exon 2 and NM_000781.3(*CYP11A1*):c.835del (p.Ile279Tyrfs*10) in exon 5. To date, only around 50 cases with *CYP11A1* pathogenic variants have been reported worldwide. We believe this is the first described case of a newborn with severe, classic P450scc deficiency in Poland. Conclusions: CYP11A1 (P450scc) deficiency is a rare and complex disorder that leads to primary adrenal insufficiency and may present with 46, XY disorders of sex development (DSD), phenotypic variations, and associated endocrinological abnormalities. This case, along with others cited, highlights the diverse presentations of DSD in individuals with pathogenic *CYP11A1* variants. Optimal management necessitates a multidisciplinary approach by a specialized DSD team. Gonadectomy is a key consideration to decrease the teratogenic risk associated with intra-abdominal gonadal tissue.

## 1. Introduction

Congenital adrenal hyperplasia (CAH) is an umbrella term encompassing many genetic disorders pathophysiologically rooted in the dysfunction of the steroidogenesis pathway. While mostly associated with 21-hydroxylase deficiency (21OHD), which constitutes more than 90% of CAH cases, it can also be caused by deficiencies in 11β-hydroxylase, 3β-hydroxysteroid dehydrogenase type 2, 17α-hydroxylase/17,20-lyase, P450 oxido-reductase, steroidogenic acute regulatory protein (*StAR*), and cholesterol side-chain cleavage enzyme (P450scc) [1]. The P450scc is a mitochondrial enzyme encoded by the *CYP11A1* gene located on the long arm of the 15th chromosome [2,3]. It catalyzes the conversion of cholesterol to pregnenolone, one of the primary steps of steroidogenesis in all tissues (adrenals, gonads, and placenta) [2,3]. In contrast to congenital lipoid adrenal hyperplasia (CLAH), which is caused by variants in the *StAR*, the P450scc is essential to steroidogenesis in all human tissues, including the placenta during pregnancy [4].

Severe disruption of this protein causes the classic form of *CYP11A1* (P450scc) deficiency, with symptoms including early (neonatal) onset salt wasting crisis (hyponatremia, hyperkaliemia, failure to thrive, hypotension), adrenal crisis (hypoglycemia, failure to thrive, hypotension), activation of the hypothalamo–hypopituitary axis with raised corticotropin-releasing hormone (CRH) and adrenocorticotropic hormone (ACTH) levels (skin and mucous hyperpigmentation), female or undervirilized external genitalia in 46, XY patients, and small adrenal glands on imaging [3,5,6,7,8,9]. As presented by Buonocore et al., nonclassic (partial) *CYP11A1* (P450scc) deficiency can present at different ages throughout childhood, often with hyperpigmentation, ketotic hypoglycemia, prolonged illness with infections, and evidence of mildly impaired mineralocorticoid secretion [5,6].

So far, to the best of our knowledge, 48 cases of P450scc deficiency and 38 pathogenic variants of *CYP11A1* have been described (Table 1).

Although in many countries, including Poland, newborn screening programs (NSP) based on 17-OH-progesterone (17-OHP) immunoassays on dried blood spots have been conducted since 2017, some of the scarcer CAH types, including P450scc deficiency, remain undetected by NSP [8]. This poses a threat of misdiagnosis by erroneously excluding CAH, which could lead to a potentially lethal state of acute adrenal insufficiency. Here, we present the case of an infant with two frameshift pathogenic variants—NM_000781.3(*CYP11A1*):c.358del (p.Arg120Aspfs*18) in exon 2 and NM_000781.3(*CYP11A1*):c.835del (p.Ile279Tyrfs*10) in exon 5 in the *CYP11A1* gene—causing primary adrenal insufficiency, early salt-wasting and adrenal crisis, and complete 46, XY sex reversal. Although reported as pathogenic, one of the variants detected in our patient, NM_000781.3(*CYP11A1*):c.358del (p.Arg120Aspfs*18) in exon 2, has not yet been clinically described, while the second, NM_000781.3(*CYP11A1*):c.835del (p.Ile279Tyrfs*10) in exon 5, was previously reported [10,11,12].

**Table 1:** List of reported patients with P450scc deficiency. Legend: ND—no data, N—no, Y—yes, DSD—disorder of sex differentiation, TART-testicular adrenal rest tumor, HELLP-H-hemolysis, EL-elevated liver enzymes, LP-low platelet count, and hCG-human chorionic gonadotropin.

Ref.	Patient	Nucleotide Position According to Ref. NM_000781.3 (*CYP11A1*)	Protein Effect	Homozygous/ Heterozygous	Onset of Adrenal Insufficiency/ Failure	Karyotype	Genitalia	Maternal Obstetric History	Gonadectomy	Growth Hormone	Adrenals Imaging
[13]	1	1. c.809_814dup; 2. wild type	1. p.Asp271_Val272ins GlyAsp 2. wild type	Heterozygous	4 years	46, XY	Female (clitoromegaly, no labial fusion, separate vaginal and urethral openings, bilateral inguinal masses)	0 miscarriages, full term pregnancy	ND	ND	normal-sized
[14]	2	1. c.566C>T 2. c.1057C>T	1. p.Ala189Val 2. p.Arg353Trp	Heterozygous	7 months	46, XX	Normal female	0 miscarriages, full term pregnancy	ND	ND	ND
[10]	3	1. c.835del 2. c.835del	1. p.Ile279Tyrfs*10 2. p.Ile279Tyrfs*10	Homozygous	9 days	46, XY	Normal female	2 prior miscarriages, born premature at 31 weeks	ND	N	ND
[15]	4	1. c.1076C>T 2. c.1076C>T	1. p.Ala359Val 2. p.Ala359Val	Homozygous	1 y 9 months	46, XY	Female (DSD, separate vaginal and urethral openings, bilateral inguinal masses)	2 prior miscarriages, full term pregnancy	Y—normal testicular tissue for age, seminiferous tubules and Sertoli cells, albeit without Leydig cells and germ cells	ND	ND
[11]	5	1. c.422T>G 2. c.1244T>A	1. p.Leu141Trp 2. p.Val415Glu	Heterozygous	1 day after birth	46, XY	Female (DSD, bilateral Abdominal masses)	0 miscarriages, full term pregnancy	Y—at 8 years of age,	ND	ND
[11]	6	1. c.835del 2. c.835del	1. p.Ile279Tyrfs*10 2. p.Ile279Tyrfs*10	Homozygous	8 days	46, XY	Normal female	1 prior miscarriage, low serum estriol and high hCG, full term	Y—bilateral atrophic testes, one contained Sertoli cell adenoma	ND	ND
[16]	7	1. c.665T>C 2. c.665T>C	1. p.Leu222Pro 2. p.Leu222Pro	Homozygous	9 years	46, XY	ambiguous genitalia/male (hypospadias and cryptorchidism)	ND/second twin (phenotypically normal female) died at the age of 2.5 y, after an unknown illness presenting with weakness and cyanosis	ND	ND	normal-sized
[12]	8	1. c.806C>T 2. c.835del	1. p.Ala269Val 2. p.Ile279Tyrfs*10	Heterozygous	9 years	46, XY	Male (micropenis, bifid scrotum, cryptorchidism)	ND	ND	ND	small, with stippled calcifications
[12]	9	1. c.806C>T 2. c.835del	1. p.Ala269Val 2. p.Ile279Tyrfs*10	Heterozygous	12 months	46, XX	Normal female	ND	ND	ND	small, with stippled calcifications
[17]	10	1. c.1351C>T 2. c.1351C>T	1. p.Arg451Trp 2. p.Arg451Trp	Homozygous	2 years 10 months	46, XY	Normal male	0 miscarriages, full term pregnancy	ND	ND	normal-sized
[17]	11	1. c.1351C>T 2. c.1351C>T	1. p.Arg451Trp 2. p.Arg451Trp	Homozygous	1 year 3 months	46, XY	Normal male	0 miscarriages, full term pregnancy	ND	ND	normal-sized
[18]	12	1. c.1078C>T 2. c.1213C>T	1. p.Arg360Trp) 2. p.Arg405*	Heterozygous	15 days	46, XY	DSD—Micropenis, penoscrotal hypospadias, mild cryptorchidism	0 miscarriages, full term pregnancy	ND	ND	normal-sized
[19]	13	1. c.694C>T 2. c.694C>T	1. p.Arg232* 2. p.Arg232*	Homozygous	Neonatal period	46, XX	Normal female	2 prior miscarriages, post-term labor	ND	ND	not found
[19]	14	1. c.694C>T 2. c.644T>C	1. p.Arg232* 2. p.Phe215Ser	Heterozygous	1.2 years	46, XY	Small penis	0 miscarriages, full term pregnancy (twin)	ND	ND	small
[19]	15	1. c.694C>T 2. c.644T>C	1. p.Arg232* 2. p.Phe215Ser	Heterozygous	14 months, died at 6.3 years	ND	Normal female	0 miscarriages, full term pregnancy (twin)	ND	ND	ND
[19]	16	1. c.694C>T 2. c.644T>C	1. p.Arg232* 2. p.Phe215Ser	Heterozygous	4.75 years	46, XY	Normal male	0 miscarriages, full term pregnancy	ND	ND	ND
[19]	17	1. c.694C>T 2. c.644T>C	1. p.Arg232* 2. p.Phe215Ser	Heterozygous	1.5 years	46, XX	Normal female	0 miscarriages, full term pregnancy	ND	ND	small
[19]	18	1. c.694C>T 2. c.694C>T	1. p.Arg232* 2. p.Arg232*	Homozygous	Neonatal period	46, XX	Normal female	2 prior miscarriages, full term pregnancy	ND	ND	ND
[19]	19	1. c.358C>T 2. c.358C>T	1. p.Arg120* 2. p.Arg120*	Homozygous	Neonatal period	46, XX	Normal female	0 miscarriages, 37 weeks pregnancy, hyperemesis	ND	ND	normal-sized
[20]	20	1. c.858G>A 2. c.858G>A	1. p.Trp286* 2. p.Trp286*	Homozygous	1 day after birth	46, XY	Normal female	3 prior miscarriages, 36 weeks pregnancy	ND	ND	enlarged
[21]	21	1. c.412G>A 2. c.508C>G	1. p.Gly138Arg 2. p.Leu170Val	Heterozygous	few hours after birth	46, XX	Normal female	36 weeks of pregnancy, recurrent bleeding from the 3rd month	ND	ND	left—hypoplastic; right— not apparent
[22]	22	1. c.1351C>T 2. c.1351C>T	1. p.Arg451Trp 2. p.Arg451Trp	Homozygous	1–6 years	46, XX	Normal female	ND	ND	ND	ND
[22]	23	1. c.1351C>T 2. c.1351C>T	1. p.Arg451Trp 2. p.Arg451Trp	Homozygous	1–6 years	46, XX	Normal female	ND	ND	ND	ND
[22]	24	1. c.1351C>T 2. c.1351C>T	1. p.Arg451Trp 2. p.Arg451Trp	Homozygous	1–6 years	46, XX	Normal female	ND	ND	ND	ND
[22]	25	1. c.1351C>T 2. c.1351C>T	1. p.Arg451Trp 2. p.Arg451Trp	Homozygous	1–6 years	46, XX	Normal female	ND	ND	ND	ND
[22]	26	1. c.1351C>T 2. c.1351C>T	1. p.Arg451Trp 2. p.Arg451Trp	Homozygous	1–6 years	46, XX	Normal female	ND	ND	ND	ND
[22]	27	1. c.1351C>T 2. c.1351C>T	1. p.Arg451Trp 2. p.Arg451Trp	Homozygous	1–6 years	46, XY	Normal male	ND	ND	ND	ND
[22]	28	1. c.1351C>T 2. c.1351C>T	1. p.Arg451Trp 2. p.Arg451Trp	Homozygous	1–6 years	46, XY	Normal male	ND	ND	ND	ND
[22]	29	1. c.1351C>T 2. c.1351C>T	1. p.Arg451Trp 2. p.Arg451Trp	Homozygous	1–6 years	46, XY	Normal male	ND	ND	ND	ND
[22]	30	1. c.1351C>T 2. c.1351C>T	1. p.Arg451Trp 2. p.Arg451Trp	Homozygous	1–6 years	46, XY	micropenis and cryptorchidism	ND	ND	ND	ND
[23]	31	1. c.940G>A 2. c.425+1G>A	1. p.Glu314Lys 2. p.?	Heterozygous	3 years	46, XY	male; hypospadias	34 weeks pregnancy; HELLP	ND	ND	normal-sized
[24]	32	1. c.940G>A 2. c.1393C>T	1. p.Glu314Lys 2. p.Arg465Trp	Heterozygous	3 years	46, XY	female, male gonads in the inguinal region	full term pregnancy	ND	ND	normal-sized
[24]	33	1. c.940G>A 2. c.1393C>T	1. p.Glu314Lys 2. p.Arg465Trp	Heterozygous	no adrenal insufficiency (diagnosis due to family history)	46, XY	male, perineal hypospadias	ND	ND	ND	ND
[24]	34	1. c.940G>A 2. c.1393C>T	1. p.Glu314Lys 2. p.Arg465Trp	Heterozygous	4 years	ND	female; small uterus	full term pregnancy	ND	ND	normal sized
[24]	35	1. c.940G>A 2. c.359G>A	1. p.Glu314Lys 2. p.Arg120Gln	Heterozygous	3 years and 8 months	46, XX	normal female; normal-sized ovaries	full term pregnancy	ND	ND	normal
[25]	36	1. c.235G > A 2. wild type	1. p.Val79Ile 2. wild type	Heterozygous	3 months	46, XY	normal male	full term pregnancy	ND	ND	normal-sized
[25]	37	1. c.235G > A 2. wild type	1. p.Val79Ile 2. wild type	Heterozygous	few hours after birth	ND	ND	ND	ND	ND	ND
[25]	38	1. c.235G > A 2. wild type	1. p.Val79Ile 2. wild type	Heterozygous	no adrenal insufficiency—according to the patient—a failure to thrive during the neonatal period (diagnosis due to CAH in patient’s children in his 50s)	46, XY	normal male	full term pregnancy	ND	ND	ND
[26]	39	1. c.1236 + 5G > A 2. c.1236 + 5G > A	1. p.? 2. p.?	homozygous	15 months	47, XXY	normal female, mildly hypoplastic testes	36 weeks of pregnancy	Y—at 22 months, normal testicular tissue with 47, XXY	ND	ND
[27]	40	1. c.1351C>T 2. c.1351C>T	1. p.Arg451Trp 2. p.Arg451Trp	Homozygous	11 years	46, XY	normal male	ND	ND	ND	ND
[28]	41	1. c.790_802del 2. c.940G>A	1. p.Lys264Leufs*5 2. p.Glu314Lys	Heterozygous	3.7 years	46, XY	normal male	full term pregnancy	Y—at 34 years—TART	N	ND
[28]	42	1. c.790_802del 2. c.940G>A	1. p.Lys264Leufs*5 2. p.Glu314Lys	Heterozygous	no adrenal insufficiency—diagnosis due to CAH in patient’s brother at 9 years	46, XY	normal male	full term pregnancy	N	N	ND
[28]	43	1. c.790_802del 2. c.940G>A	1. p.Lys264Leufs*5 2. p.Glu314Lys	Heterozygous	no adrenal insufficiency—diagnosis due to CAH in patient’s brother at 10 years	46, XY	normal male	full term pregnancy	N	N	ND
[29]	44	1. c.566C > T 2. c.1236 + 5G > A	1. p.Ala189Val 2. p.?	Heterozygous	10 months	46, XY	normal female	0 miscarriages, full term pregnancy	Y—typical testicular tissue	ND	ND
[30]	45	1. c.940G>A 2. c.835del	1. p.Glu314Lys 2. p.Ile279Tyrfs*10	Heterozygous	3.6 years	46, XY	normal male	0 miscarriages, full term pregnancy	ND	ND	bilaterally atrophic
[30]	46	1. c.940G>A 2. c.835del	1. p.Glu314Lys 2. p.Ile279Tyrfs*10	Heterozygous	9 years	46, XY	normal male	0 miscarriages, full term pregnancy	ND	ND	bilaterally normal adrenal glands without evidence of hyperplasia
[30]	47	1. c.940G>A 2. c.835del	1. p.Glu314Lys 2. p.Ile279Tyrfs*10	Heterozygous	5 years	46, XY	normal male	0 miscarriages, full term pregnancy	ND	ND	ND
[30]	48	1. c.940G>A 2. c.835del	1. p.Glu314Lys 2. p.Ile279Tyrfs*10	Heterozygous	4.8 years	46, XY	normal male	0 miscarriages, full term pregnancy	ND	ND	ND

## 2. Case Presentation

The female infant was born to healthy, non-consanguineous Caucasian parents after a first pregnancy and 38 weeks of gestation. The pregnancy was complicated by gestational hypertension and increased nuchal translucency. The Apgar score was 4/4/5/5 at the 1st/3rd/5th, and 10th minute, respectively, and the birth weight was 3400 g. The patient was born with primary apnea, hypotonia, and a heart rate of 100/min. After 10 min of infant resuscitator (Neopuff) ventilation and a fluid bolus, normal breathing appeared. In the following days, nasal continuous positive airway pressure (nCPAP) was introduced due to numerous episodes of apnea and bradycardia. On the 2nd day post-delivery, the infant developed jaundice, which was treated with phototherapy for four days. As the jaundice subsided, skin hyperpigmentation became gradually apparent. A differential diagnosis was compiled and returned negative for cytomegalovirus and Toxoplasmosis infection, hypothyroidism, alfa-1 antitrypsin deficiency, and galactosemia. Physical examination showed generalized skin hyperpigmentation, hypotonia, signs of dehydration, and normal female external genitalia. Deteriorating condition, increasing hypoglycemia, hyponatremia, and hyperkalemia, together with elevated ACTH (>1380 pg/mL), plasma renin activity (>31 ng/mL/h), and low cortisol (<5 ng/mL) prompted the suspicion of adrenal insufficiency (Table 2), which was later confirmed with a 24-h urine steroid profile analysis (Table 3). Hydrocortisone and fludrocortisone supplementation were initiated during the patient’s first week of life (Table 4). The patient remained hospitalized for the first seven weeks. Within the initial six days, the newborn was transferred from a local hospital to a regional intensive care unit and subsequently to a tertiary Neonatal Intensive Care Unit (NICU), where she spent an additional six weeks. After discharge, the patient was reevaluated weekly during the first month, biweekly in the second month, and then monthly until her first birthday. From age 1 to 2 years, follow-ups occurred every 2–3 months and every 3–4 months thereafter. The patient is currently 7.5 years old.

**Figure 1:**
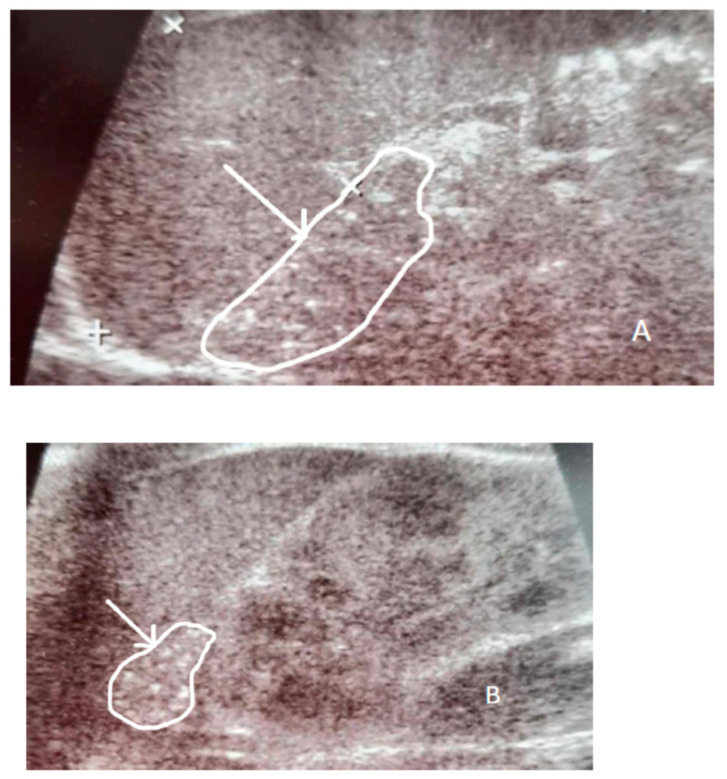
Ultrasound scans showing calcifications in (**A**)—right adrenal gland and (**B**)—left adrenal gland (white arrows, white line encircles the adrenal glands).

**Figure 2:**
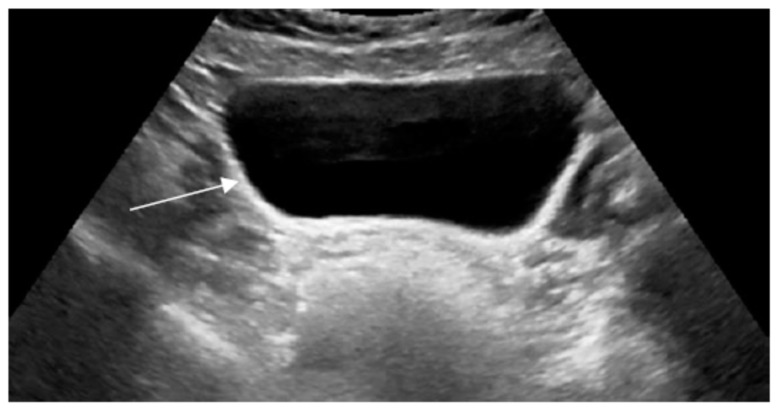
Ultrasound examination scan of the lower abdomen and pelvis. Imaging assessment within the first month of life demonstrated the absence of the uterus and ovaries (not visible below the urinary bladder). White arrow indicates the urinary bladder.

Ultrasound assessment in the first month of life revealed hepatosplenomegaly and small hyperechogenic adrenal glands with multiple calcifications. The uterus and ovaries, however, were absent (Figure 1 and Figure 2).

Additionally, echocardiography, performed in the first month of life, revealed left ventricular hypertrophy and mild pericardial effusion, although these findings were clinically asymptomatic. Left ventricular hypertrophy resolved by 1.5 years of age, as confirmed by follow-up echocardiography.

At 9 months of age, an ultrasound examination showed signs of nephrocalcinosis, which was probably linked to high doses of corticosteroids administered after birth and continued until the age of 6 months. In order to prevent further kidney injury, magnesium citrate supplementation was recommended. The patient remains under nephrological surveillance for nephrocalcinosis.

At the age of 3, the patient experienced an adrenal crisis characterized by unconsciousness and severe hypoglycemia during a febrile illness, necessitating urgent hospital admission.

Due to the negative result of the screening test for congenital adrenal hyperplasia (low 17-OH-progesterone concentration) (Table 2 and Table 3), molecular analysis for possible causes of primary adrenal insufficiency (PAI) was conducted. Chromosome analysis identified a 46, XY karyotype, which suggested conditions presenting with both PAI and 46 XY disorders of sex development (DSD).

Notably, assessment of the gonadal axis during the minipuberty period revealed low-normal levels of luteinizing hormone (LH), follicle-stimulating hormone (FSH), and testosterone (Table 2). Additionally, we evaluated hormones produced by Sertoli cells, including anti-Müllerian hormone (AMH) and inhibin B. AMH levels were elevated compared to normal female infants but lower than expected for male infants (Table 2). Nonetheless, the AMH level was evidently sufficient during fetal development to inhibit uterine formation in this patient. Inhibin B levels were within the low-normal range. Collectively, these hormonal assessments suggested the presence of intra-abdominal male gonads.

Wolman’s disease, lysosomal storage diseases, and variants in the *StAR*, *SF-1*, *SOX-9*, and *DAX-1* genes were excluded. Antley-Bixler syndrome (P450 oxidoreductase deficiency) was ruled out based on a 24 h urine steroid profile and whole exome sequencing.

Due to financial constraints, whole exome sequencing (WES) was conducted only at the age of 6. The patient’s genomic DNA was extracted from the whole blood sample using Prepito TM, and the sequencing exome library was prepared according to CeGaT Exome Enrichment (Twist Bioscience). The enriched DNA libraries were sequenced by the Il-lumina NovaSeq 6000 instrument, 2 × 100 bp. All procedures for exome sequencing were conducted by CeGaT (Germany). Raw sequencing reads were mapped to the human reference genome GRCh37 assembly using BWA MEM (bwa-mem2.avx2 mem 0.7.17-r1188) [31]. Duplicates were removed using biobambam2 version 2.0.183 [32]. Variants were called using HaplotypeCaller (GATK v4.2.6.1 [33,34]) and FreeBayes v1.3.2 [35], and they were named using Variant Effect Predictor (VEP109) [36]. The variants were classified according to the American College of Medical Genetics and Genomics (ACMG) recommendations [37]. The presence of the variant in control populations was checked in 1000Genomes [38] and gnomAD (Broad Institute) [39]. The in silico splicing analysis was performed using algorithms embedded in Alamut Visual Plus software (Sophia Genetics https://www.sophiagenetics.com/ufaq-category/alamut-visual-plus/, date 27 September 2024), i.e., SpliceSiteFinder-like, MaxEntScam, NNSPLICE, and GeneSplicer and SpliceAI—[40].

**Figure 3:**
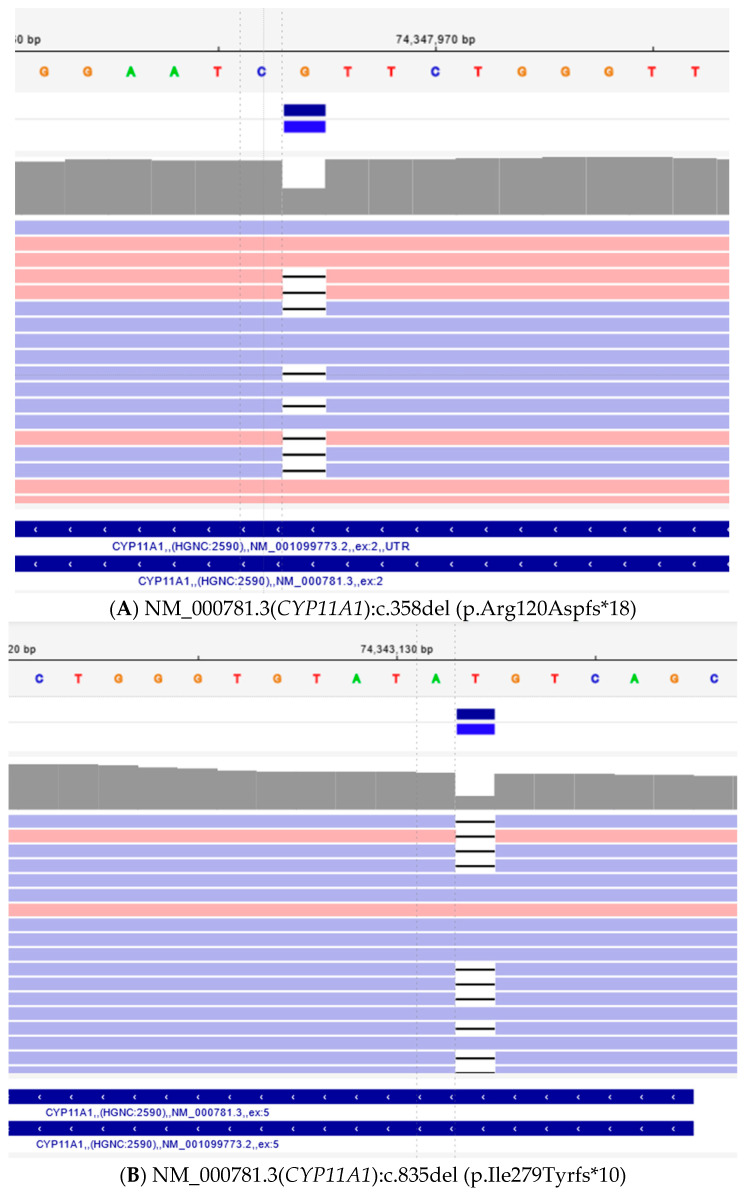
Identification of two pathogenic variants in *CYP11A1* gene. Next-generation DNA sequencing reads of the *CYP11A1* gene from the patient aligned to the reference sequence are visualized in the Integrative Genomics Viewer (IGV); (**A**)—c.358del (p.Arg120Aspfs*18) (**B**)—c.835del (p.Ile279Tyrfs*10).

**Figure 4:**
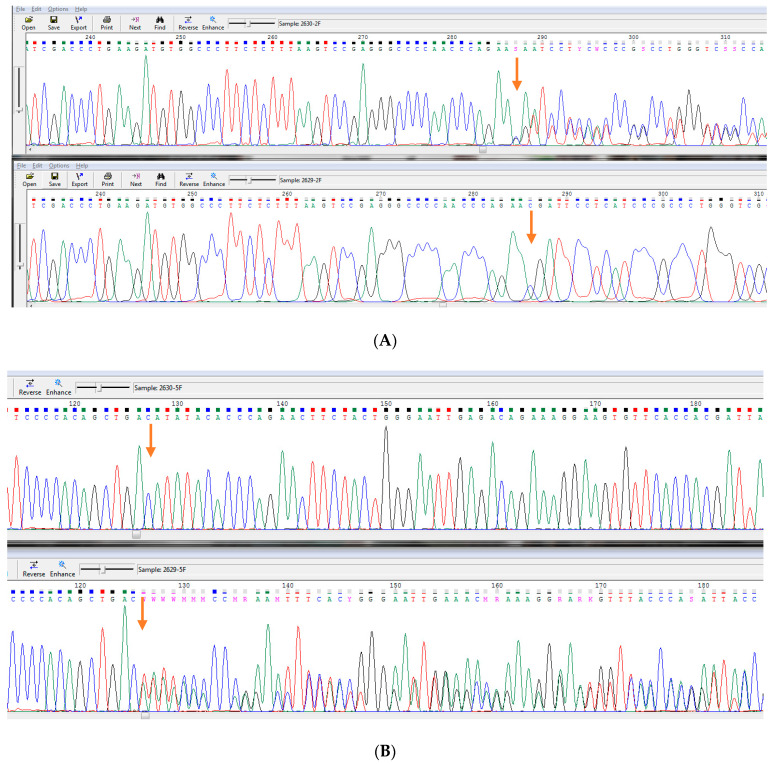
Results of automated DNA sequencing for *CYP11A1* presenting paternal origin of pathogenic variant c.358del (p.Arg120AspfsTer18) (**upper** chromatogram) and maternal origin of pathogenic variant c.835del (p.Ile279TyrfsTer10) (**bottom** chromatogram), thus confirming that our patient inherited pathogenic variants from both parents. (**A**) c.358del (p.Arg120AspfsTer18): Paternal origin of heterozygous pathogenic variant c.358del (p.Arg120AspfsTer18). Maternal sequence is normal. (**B**) c.835del (p.Ile279TyrfsTer10): Paternal sequence is normal, Maternal origin of heterozygous pathogenic variant c.835del (p.Ile279TyrfsTer10). Legend: paternal chromatogram is in upper line, maternal chromatogram is in bottom line. Arrows indicate the localization of the variants.

Whole exome sequencing revealed two heterogenic pathological variants, one for each copy of the *CYP11A1* gene, which confirmed P450scc deficiency in this patient. Next-generation DNA sequencing reads of the *CYP11A1* gene from the patient are presented in Figure 3. Figure 4 presents the results of automated DNA sequencing for *CYP11A1*, demonstrating the paternal origin of the pathogenic variant NM_000781.3(*CYP11A1*):c.358del (p.Arg120Aspfs*18) and the maternal origin of the pathogenic variant NM_000781.3(*CYP11A1*):c.835del (p.Ile279Tyrfs*10). This confirms that our patient inherited pathogenic variants from both parents.

**Figure 5:**
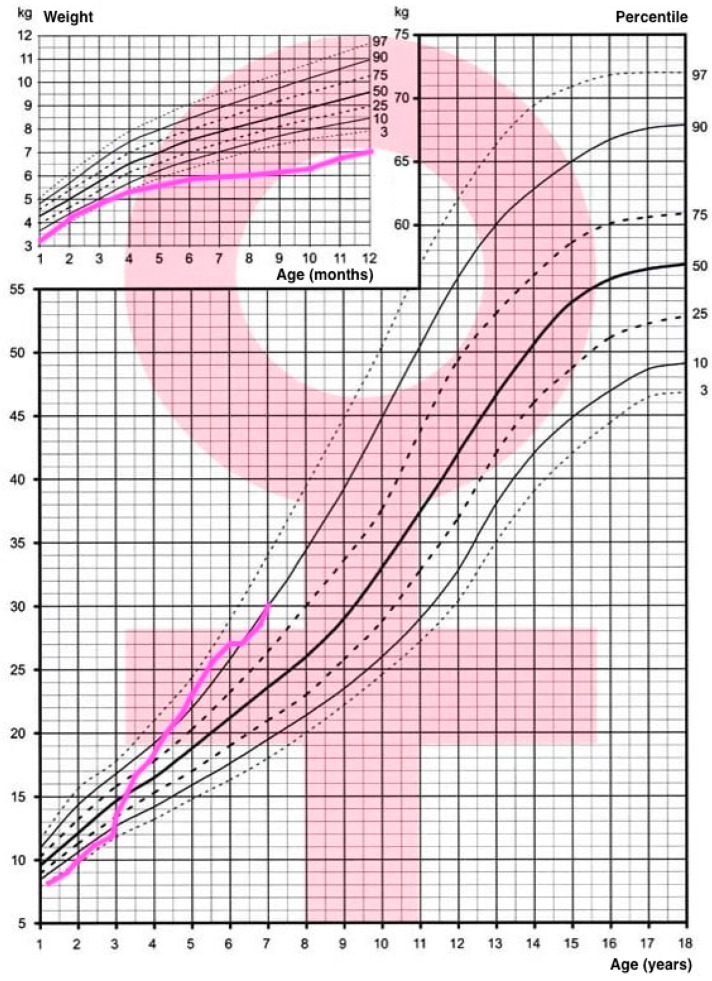
Weight percentile chart by Palczewska and Niedzwiedzka, weight trajectory marked in pink [41].

**Figure 6:**
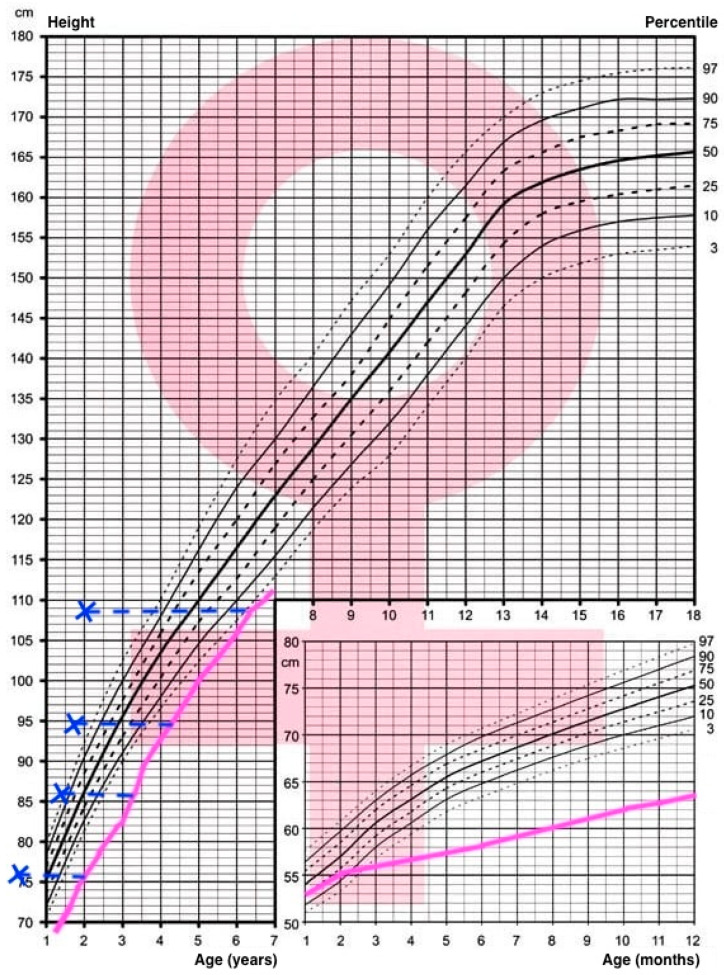
Height percentile chart by Palczewska and Niedzwiedzka, height trajectory marked in pink, bone age marked with blue crosses [41].

The patient presents complete XY sex reversal with normal prepubertal female external genitalia. From birth, the patient and her parents have been under the care of a Disorder of Sex Development (DSD) team, consisting of a psychologist, urologist, geneticist, pediatrician, and endocrinologist. The patient’s growth had followed a linear trend, staying below the 3rd percentile, while the weight stayed below the 3rd percentile until the age of 3 when it increased and later oscillated between the 75th and 97th percentile, as presented on the national Polish growth charts by Palczewska and Niedzwiedzka (Figure 5 and Figure 6) [41]. A delayed bone age has also been observed (at the age of 4.5 years, the bone age, according to a wrist x-ray, was between 1.5 and 2 years), which gives hope for implementing growth hormone replacement therapy in the future.

The patient’s phenotype is characterized by short stature (−2.1 SD), central obesity (154% of due weight), dysmorphic macrocephaly, together with shortened upper and lower extremities. Characteristic facial features include a narrow middle section of the face, small ears with thick helix, and fleshy upturned lobules. Despite some minor setbacks during the first year of life (sitting at 9 months, walking at 14 months) and rehabilitation during infancy due to hypotonia, the patient currently presents only minor disruptions in gross motor skills, while fine motor skills are not impaired.

An important prospective consideration is performing a gonadectomy prior to initiating growth hormone (GH) therapy in our patient. All GH levels during the glucagon stimulation test were below the diagnostic threshold, indicating growth hormone deficiency (10 ng/mL). However, the patient needs a second confirmatory test (with arginine or clonidine), which is currently postponed due to a family request.

## 3. Discussion

We report the first case of congenital adrenal hyperplasia and complete sex reversal due to severe P450scc deficiency with the unique clinical dysmorphic phenotype. The reported patient presented with short stature, central obesity, dysmorphic macrocephaly, together with shortened upper and lower extremities. Characteristic facial features included a narrow middle section of the face, small ears with thick helix, and fleshy upturned lobules. To the best of our knowledge, the dysmorphic features that were observed in our patient have not been described in other patients with P450scc so far. The delayed bone age (delayed ossification) could also point to the role of P450scc in chondrogenesis. However, the most probable explanation of delayed bone age could be growth hormone deficiency in our patient. Some of this patient’s features could be typical for individuals with growth hormone deficiency prior to growth hormone therapy (midfacial hypoplasia, small hand and feet, central distribution of fat tissue), which is suspected in our patient.

This is also the first report of classic, severe P450scc deficiency caused by a pathogenic variant in the *CYP11A1* gene in a newborn in Poland. One of the variants detected in our patient, NM_000781.3(*CYP11A1*):c.358del (p.Arg120Aspfs*18), although reported as pathogenic, has not yet been clinically described, while the second, NM_000781.3(*CYP11A1*):c.835del (p.Ile279Tyrfs*10), was previously reported in both homozygous and compound heterozygous patients [10,11,12].

Hiort et al. reported a case of a 46, XY infant with complete sex reversal and p.Ile279Tyrfs*10 homozygous variant, who, similarly to our patient, developed signs of adrenal insufficiency on the 9th day of life and respiratory failure needing assisted ventilation [10]. In the aforementioned case, ultrasound and magnetic resonance imaging failed to demonstrate gonads or adrenal tissue, and no uterus was seen [10]. The second described case, by Kim et al., presented a patient with a 46, XY karyotype, complete sex reversal, and heterozygous p.Ile279Tyrfs*10 pathogenic variant, who showed signs of adrenal insufficiency on the 6th day of life [11]. She also had hypogonadotropic hypogonadism, a tethered spinal cord due to a lipoma, hyperlordosis, strabismus, central hypothyroidism, subnormal GH secretion, and short stature [11]. On imaging, no gonads, internal reproductive structures, or adrenals were identified; however, in a performed gonadectomy, bilateral atrophic testes were found, one of which contained a small Sertoli cell adenoma [11]. Similarly to both of these cases, we detected neither uterus nor gonads during abdominal ultrasonography and MRI. Our patient awaits the decision to perform a diagnostic laparoscopy in order to remove abdominal gonadal tissue, which could carry a teratogenic risk.

The third study, by Sahakitrungruang et al., described two siblings with compound heterozygous p.Ile279Tyrfs*10 pathogenic variant, late-onset adrenal insufficiency resembling Addison disease, and minimally disordered sexual development [12]. The first was, at the time of presentation, a 9-yr-old 46, XY male with micropenis, severe hypospadias, bifid scrotum, cryptorchidism, and hyperpigmentation [12]. The second was a 46, XX female with normal prepubertal genitalia and mild hyperpigmentation [12]. In both patients, abdominal computed tomographic imaging showed slightly smaller adrenals with stippled calcifications, suggesting cirrhotic, end-stage fat deposition reported in nonclassical lipoid CAH due to the StAR variants [12,42]. Similarly, in the abdominal USG examination, our patient had small hyperechogenic adrenal glands with multiple calcifications.

In the first two previously described cases, mothers had a history of miscarriage and low maternal plasma estriol during pregnancy, while the mother of our patient did not report any history of obstetric complications [10,11]. The only irregularities reported during pregnancy were gestational hypertension and nuchal translucency. The third study provided no data on the mother’s obstetric history [12]. The patient described by Hiort et al. was born preterm at 31 weeks of gestation, while our patient and the one described by Kim et al. were both carried to term and born on the 38th and 39th weeks, respectively [10,11].

The NM_000781.3(*CYP11A1*):c.835del (p.Ile279Tyrfs*10) variant of *CYP11A1* gene was tested in vitro and proven to carry no enzymatic activity [11]. Our novel variant NM_000781.3(*CYP11A1*):c.358del (p.Arg120Aspfs*18) lies in the second exon of the *CYP11A1* gene and causes a frameshift mutation on the 120th amino acid (p.Arg120fs). As the whole protein encompasses a sequence of 521 amino acids, this pathogenic variant has the capacity to render the synthesized protein devoid of its original activity, due to enormous changes in the amino acid sequence. It is corroborated by a predictive analysis on the “Franklin by genoox” site (https://franklin.genoox.com, accessed on 5 September 2024), which indicates a pathogenic effect on P450scc [9]. It further classifies the pathogenic variant’s effect on protein as very strong (PVS1) due to producing a null variant allele, which causes loss of function, a known mechanism of disease, as 18 pathogenic null variants have already been reported for this gene (5 of which were also reported in the 2nd exon). Furthermore, it predicts a pathogenic moderate risk (PM2) due to an extremely low frequency in population databases. Finally, a reputable source recently reported this variant as pathogenic, providing the PP5 criterion. All in all, the variant meets the requirements for pathogenicity set by the ACMG (one PVS1 + one PM2 and one PP5) [37].

This, however, poses a genotypic/phenotypic inconsistency. If both of the alleles produced non-functional proteins, then no placental progesterone could have been synthesized. As progesterone is necessary in order to maintain pregnancy, this could point to an alternative source of progesterone during pregnancy. Earlier papers floated the idea of prolonged biosynthetic activity of the maternal corpus luteum or an alternative enzymatic pathway producing progesterone during pregnancy [2]. No definitive conclusions have been, however, established.

Finally, similarly to the patient described by Kim et al., who carries a heterozygous p.Ile279Tyrfs*10 pathogenic variant, our patient also presents with subnormal GH secretion and short stature [11]. This prompts the issue of introducing GH therapy in order for the patient to achieve optimal final height. However, as growth hormone supplementation may potentially raise the teratogenic risk, which is already increased in the abdominally located gonadal tissue of patients with P450scc, as described in previous cases, firstly, one should consider gonadectomy. We have found six incidences of gonadectomy performed in patients with P450scc [11,15,26,28,29] (Table 1). In the case reported by al Kandari et al., the removed gonads contained normal testicular tissue for age, seminiferous tubules, and Sertoli cells, albeit without Leydig cells and germ cells [15]. Of the two patients described by Kim et al., one had bilateral atrophic testes, one containing Sertoli cell adenoma, while the other had no description of histopathologic findings [11]. Kallali et al. described an orchidectomy performed on a 25-year-old patient, which found a testicular adrenal rest tumor (TART) in the removed left testicle [28]. Finally, Kolli et al. reported peripubertal evidence of TART detected by ultrasound in two male patients [30]. In other described gonadectomies, normal testicular tissue was found.

The limitation of this study is the fact that we have not yet performed a gonadectomy. In our patient, no gonadal tissue has been observed either on ultrasonographic or magnetic resonance imaging. However, a diagnostic laparoscopy with possible gonadectomy is under consideration. The strengths of this study should include the presentation of dysmorphic features not previously reported in *CYP11A1* (P450scc) deficiency.

## 4. Conclusions

*CYP11A1* (P450scc) deficiency is a rare and complex disorder that leads to primary adrenal insufficiency and may present with 46, XY disorders of sex development (DSD), phenotypic variations, and associated endocrinological abnormalities. This case, along with others cited, highlights the diverse presentations of DSD in individuals with the pathogenic *CYP11A1* variants. Optimal management necessitates a multidisciplinary approach by a specialized DSD team. Gonadectomy is a key consideration for decreasing the teratogenic risk associated with intra-abdominal gonadal tissue.

## Figures and Tables

**Table 2:** Hormonal concentrations in the first weeks of life (minipuberty). Legend: PRA-plasma renin activity; AMH-anti-Müllerian Hormone.

Day of Life	5th	7th	13th	20th	23rd
17-OHP [ng/mL] 0.02–0.84	0.03			0.01	
ACTH [pg/mL] 5–40		1380			
cortisol [ng/mL] 30–160		<5.0			
PRA [ng/mL/h] < 35		>31.0			
FSH [mIU/mL] ♂ 2.50 ± 1.51 ♀ 7.07 ± 5.92					0.6
LH [ mIU/mL] ♂ 2.86 ± 1.51 ♀ 0.46 ± 0.25					0.14
Testosterone [ng/mL] 0.1–0.8					0.17
AMH [ng/mL] ♀ < 4.0 ♂ 35–135					12.21
Inhibin B [pg/mL] 25–325					60
Aldosteron [pg/mL] 70–900			<7.6		

**Table 3:** Twenty-four hour urine steroid profile. Legend: An—Androsterone; Et—Etiocholanolone; 11-OAN/ET—11-ketoandrosteron/etiocholanolone; 11-OHAN—11-hydroxy-androsterone; 11OHET—11-hydroxy-etiocholanolone; DHA—Dehydroepiandrosterone; 5-AND—5-androstendiole; 16a-OHDHA—16a-Hydroxy-DHA; An-3-ol-5 androstentriole; 5PT—5-Pregnenetriol; 16-OHPN—16 alfa-hydroxy-pregnenolone; 17OHPN—17-hydroxy-pregnanolone; PT—Pregnanetriol; PTN—Pregnanetriolone; PD—Pregnanediol; E1—estron; E2—estradiol; E3—estriol; THS—Tetrahydro-11-deoxycortisol; THDOC—Tetrahydro-11-deoxycorticosterone; THA—Tetrahydro-11-dehydrocorticosterone; THB—Tetrahydrocorticosterone; THAldo—3a,5b-Tetrahydroaldosterone; THE—Tetrahydrocortisone; THF—Tetrahydrocortisol; a-CTN—a-Cortolone; b-CTN—b-Cortolone; a-CT—a-Cortol; b-CT—b-Cortol; E—Cortisone; F—Cortisol; 6b-OHF—6b-Hydroxycortisol, 20a-DHF—20alfa/beta-dihydrocortisol.

Steroid Profile	Value [µg/24h]	Norm
*AN*	3.5	[1–10]
*ET*	1.6	[1–5]
*11-OAN/ET*	0.7	[5–20]
*11-OHAN*	2	[2–20]
*11-OHET*	0.5	
*ET/AN*	0.5	
*DHA*	1.5	[1–10]
*5-AND*	5.2	[1–10]
*16a-OHDHA*	1.8	[135–500]
*An-3-ol*	0.9	[40–600]
*5-PT*	1.9	[2–20]
*16-OHPN*	1.2	[110–495]
*17-OHPN(5beta)*	1.1	[4–19]
*17-OHPN(5alfa)*	0.1	
*PT*	2.8	[5–21]
*PTN*	0.3	[0–5]
*PD*	3	[2–20]
*E1*	0	
*E2*	0	
*E3*	0	
*THS*	0.2	[1–3]
*THDOC*	0	
*THA*	0	[5–30]
*allo-THA*	0	[15–90]
*THB*	0	
*allo-THb*	0	
*THAldo*	0.3	[4–12]
*THE*	1.9	[38–408]
*THF*	0.8	
*allo-THF*	0.5	
*THF/allo-THF*	0.8	
*THF+allo-THF/THE*	0.9	
*a-CTN*	1	[20–100]
*b-CTN*	0.8	[20–100]
*b-CT*	0.5	[5–20]
*a-CT*	1.4	[5–20]
*E*	0.4	[5–20]
*F*	0.8	[3–20]
*F/E*	2.1	
*6b-OHF*	0	
*20a-DHF*	0	

**Table 4:** Hydrocortisone (HC) and fludrocortisone (FC) dosing regimens over time.

**Hydrocortisone Dosing**
First week of life: 42.8 mg/m^2^/day intravenously (3 doses of 3 mg HC per day). Second week of life: 57.1 mg/m^2^/day orally (3 doses of 4 mg HC per day). Third week of life: 57.1 mg/m^2^/day intravenously due to a rotavirus infection (3 doses of 4 mg HC per day). Fourth week of life: Due to clinical instability, the HC dose was increased from 85.7 mg/m^2^/day intravenously to 142 mg/m^2^/day orally, followed by a gradual taper over two weeks. Upon discharge, the patient was maintained on 3 doses of 7.5 mg HC orally per day (107 mg/m^2^/day). Outpatient adjustments: The HC dose was gradually reduced to 25.86 mg/m^2^/day at 5 months of age and to 20.8 mg/m^2^/day at 7 months of age. Current regimen (age 7.5 years): 5 mg + 2.5 mg + 2.5 mg HC daily (10.75 mg/m^2^/day).
**Fludrocortisone Dosing**
First week of life: 50 µg daily. Second week of life: Increased to 100 µg daily. Third week of life: Reduced to 50 µg daily. Fourth week of life: Increased to 75 µg daily. Discharge regimen: 50 µg daily. Current regimen (age 7.5 years): Continues on 50 µg daily.
**Sodium Chloride Supplementation**
Oral 10% sodium chloride (NaCl) supplementation began in the fourth week of life with 8 doses of 1 mL 10% NaCl administered with meals. This regimen continued until the end of her first year and was then discontinued.

## Data Availability

The original contributions presented in the study are included in the article. Further inquiries can be directed to the corresponding author.
